# The Role of Time in the Structural Ordering of Poly-3-Hexylthiophene

**DOI:** 10.3390/polym17223077

**Published:** 2025-11-20

**Authors:** Ikemefuna Uba, Wisdom Jagoi, Brenden Forrest, Abdul-Majeed Hamidu, Kenneth Granderson, Emmanuel Baskerville, Lailah Outsey, Robert Birdow, Kamar Mann, Justice Ash

**Affiliations:** Department of Electrical and Computer Engineering, Hampton University, Hampton, VA 23668, USA; wisdom.jagoi@my.hamptonu.edu (W.J.); brenden.forrest@my.hamptonu.edu (B.F.); abdul.hamidu@my.hamptonu.edu (A.-M.H.); kenneth.granderson@my.hamptonu.edu (K.G.); emmanuel.baskerville@my.hamptonu.edu (E.B.); lailah.outsey@my.hamptonu.edu (L.O.); robert.birdow@my.hamptonu.edu (R.B.); kamar.mann@my.hamptonu.edu (K.M.); justiceash11@gmail.com (J.A.)

**Keywords:** polymer, P3HT, optical analysis

## Abstract

An investigation into the influence of annealing time on structural ordering of Poly-3-Hexylthiophene has been performed via analyses of absorbance data, on the premise that ordering status is reflected in optoelectronic properties. Thin films heated from 0 to 100 min were examined. It was found that 20 min annealing yields a thin film with the highest structural ordering, with density of states of 6.08 ± 0.16 × 10^38^ m^−3^ kg^−1^ and carrier density of 5.54 ± 0.14 × 10^8^ m^−3^. Samples annealed beyond 40 min exhibited optoelectronic traits midway between unannealed and 20 min annealing. The results demonstrated that during thermal treatment at a fixed temperature, the structural ordering and conformity continuously change with time, affecting the optoelectronic properties, thus emphasizing the necessity of the “time-profile” to determine the appropriate annealing time for a specific application of the polymer.

## 1. Introduction

Poly-3-Hexylthiophene (P3HT) may be considered a reference point in the polymeric semiconductor space, given its ubiquitous presence in the organic semiconductor ecosystem. This semi-crystalline, p-type semiconductor is known to have properties that depend on molecular weight, regio-randomness and regioregularity of the molecular chains. Regio-regular P3HT (rr-P3HT) with regularity ≥ 90% exhibits excellent electrical and photophysical properties [1,2,3,4]. Notwithstanding this molecular factor, processing methods and conditions largely control the observable responses of the polymer in solid form. This is influenced through chain conformation, orientation and packing, which are directly impacted by deposition process methods [5,6,7,8]. Drop-casting and spin coating provide facile solution-based techniques for P3HT thin films and device fabrications. The former suffers from irreproducibility of thickness and morphology, while the latter ensures repeatable thickness once the spin-coating system is adequately calibrated. However, the process yields films with molecular chains that are in non-equilibrium state due to the accelerated evaporation of solvent [9]. Thus, thermal treatment of the films is necessary to attain the desired responses, and therein lie the divergent conclusions.

Thermal treatment generally improves the structure, electrical and optoelectronic properties of thin films. It can cause (1) increased crystallite size that shows up in x-ray diffraction as heightened intensity at relevant crystal planes, (2) formation of crystalline region and (3) reorganization of structure or molecular chains. For P3HT, there exists an array of somewhat conflicting impacts of annealing. In a comparative study of unannealed and annealed samples, thermally improved crystallinity was the reason for increased absorption [10,11]. Contrary to this, Gurau et al. [12] found that subjecting as-cast films to the melting point of P3HT (250 °C) and allowing them to recrystallize at lower temperatures led to reorganization of chain order, which was signatured by a decrease in absorption. Films annealed at 180 °C for 12 h had better ordering, with absorption spectra well below those of as-cast films, whereas 220 °C for 1 h and 100 °C for 12 h yielded spectral intensities that were similar to those of as-cast films, thereby suggesting that they have the same molecular chain ordering. To put the controversy in perspective, we note that P3HT crystallizes in the orthorhombic structure at room temperature [1]. Across the literature, the presence of distinct crystalline phase is signatured by a “shoulder” in absorption spectrum, and ordering refers to the π-stacking [13,14,15,16]. Thus, the increased absorption after heating at 150 °C for 10 min in [4] might have been due to disorder, despite the formation of the crystalline phase. Unfortunately, no further annealing was performed to check the conclusion therein. In line with [6], Delongchamp et al. [17] attributed improved charge mobility to enhanced π-π* stacking caused by thermal-induced reorganization of the crystal structure. In the case of varied annealing temperatures, Peng et al. [5] related absorption maxima to the quasi-ordered state of the molecular chain, which suggested a temperature-moderated interconversion between the crystalline and amorphous states, as well as interchain and intrachain ordering. In addition, x-ray diffraction has been leveraged in some works to explore the impact of annealing, which revealed no correlation between crystalline size and photophysical properties [1,5,9]. This might be attributed to the observation that solution concentration determines the final crystalline size [1]. The absence of a conclusive direct correlation between annealing and crystallinity can be appreciated in the light of [5,10,12,17] and the fact that P3HT crystallizes at room temperature [1]. Hence, we can surmise that thermal treatment has little impact on crystallinity but significantly controls polymer chain orientation, conformation and packing order in P3HT and that this control is reversible and repeatable, as pointed out in [5,9]. The nature of chain orientation, conformation and packing order determines optical responses. It follows then that the degree of ordering and transformation will be registered qualitatively and quantitatively (intensity) in the absorption spectrum of a P3HT film.

In light of the above, and considering heat as a perturbing field, the question arises of whether annealing time is a significant factor in heat-induced reversible transformation. This is pertinent to the ecosystem of P3HT, because it will facilitate a systematic profiling of the polymer for specific applications within a given processing condition (solvent, concentration, etc.). In the literature, annealing time has been about one hour. If time-dependence exists, then by implication, several reorderings must have occurred within the hour; thereby obscuring structural transformations that could have been more suitable for the applications reported.

In this submission we address this question of time-dependent reordering. Here, the impact of time-dependent annealing at 80 °C is investigated by absorption spectroscopy. Optoelectronic properties extracted by this technique reveal the structural state of a material. The temperature is high enough to induce reversible ordering and well below the melting point of P3HT, thereby mitigating an undesirable phase state. This work was motivated by the need for the best annealing time for the polymeric transistors and diodes that we are developing. The scope is constrained to optoelectronic analyses as a means to judge chain ordering without recourse to crystallite size studies, since existing literature had demonstrated that a “shoulder” in UV-Vis spectrum indicates the presence of crystallinity and good ordering encourage good electrical/electronic properties [12,18,19].

## 2. Materials and Methods

**Figure 1:**
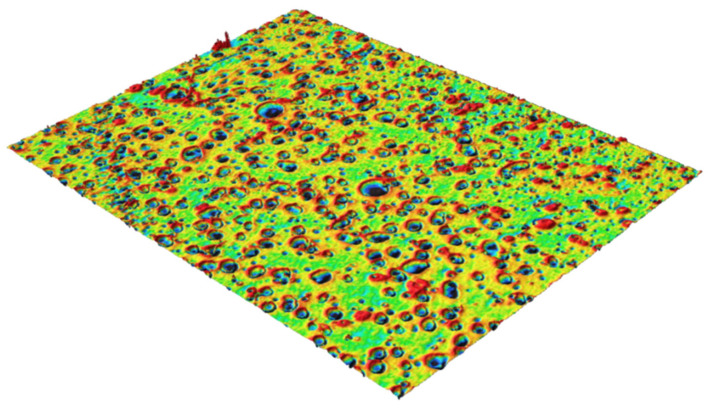
Three-dimensional surface morphology of a P3HT film from 3D optical profilometry.

Analytical grade rr-P3HT of 97.6% regioregularity and 60,150 g/mol molecular weight from Ossila Inc, Sheffield, UK was used as received. A total of 10 mg of this was dissolved in 1 mL of chloroform at ambient temperature. The solution was agitated on a Thermo Scientific U.S.A, LP vortex mixer at 1000 rpm for 5 min to induce complete dissolution and reduce clustering, yielding a brownish solution. Six 20 × 20 mm glass substrates were first immersed in acetone for an hour to degrease the surfaces and afterwards were rinsed with distilled water and dried in open-air. They were then further cleaned in Plasma cleaner at 560 mTorr and high RF for 20 min to remove surface impurities and mitigate contributions from foreign atoms to the characteristics of the polymer films. A total of 0.3 mL of the P3HT-chloroform solution was spin-deposited on each substrate at 600 rpm for 30 s using Apogee Spin Coater (Cost Effective Equipment, St. James, MO, USA). Given the high evaporation coefficient of chloroform, the spin speed and duration were adequate to remove solvents from the films, which yielded uniform very thin and translucent films. Based on literature report [9], the films were assumed to be in a non-equilibrium state and have short conjugations. Five samples were annealed at a fixed temperature of 80 °C and for different times from 20 to 100 min in 20 min intervals. We used the approach of collective heating and removal of a sample at specific time. That is, once the oven temperature stabilized at 80 °C, all the samples to be annealed were loaded together, and an annealed sample was removed sequentially at a specific time. The approach guaranteed that the annealed films were in the same external condition and simultaneously underwent the same internal process. This mitigates the vagaries inherent in the alternative method of subjecting a single sample to repeated annealing and characterization. Thus, the impact of time is the only variable external factor. The films were labeled according to the annealing time with S0 being the as-deposited (unannealed) sample. The films had thicknesses of approximately 65 nm. Prior to the final reported case, a calibration of the Apogee Spin Coater thickness yield for P3HT solution as described above was performed, using Keyence Vk-X3000 optical Profilometer (Keyence Corporation of America, Itasca, IL, USA). It showed a consistent thickness of about 65 nm^++^. The Vk-X3000 is a non-destructive profilometer that can measure thickness and map surface roughness. Figure 1 shows a sample surface.

To capture the optical response, a Shimadzu 3600 UV-Vis spectrophotometer (Shimadzu Scientific Instruments, Columbia, MD, USA) was used to record the absorbance at 330–1000 nm wavelength. The absorbance spectrum is a simple but critical response in understanding nanoscale materials, since it carries the signature of the structural states, and when carefully analyzed, reveals optoelectronic parameters.

## 3. Results and Discussion

The focus of this report is to resolve the existence (if any) of time-dependent reordering in P3HT. We assumed that such an occurrence would be carried in the optical responses and reflected in the quantitative values of the optoelectronic properties thereof. To this end, it suffices to use a single measurable optical response to extract and analyze the properties of the P3HT polymer in solid thin film with respect to annealing time. In this regard we favored the absorbance in the 330–1000 nm wavelength, which revealed the impact of annealing on optoelectronic properties and on the structural layout of the polymer.

### 3.1. Annealing and Absorbance

**Figure 2:**
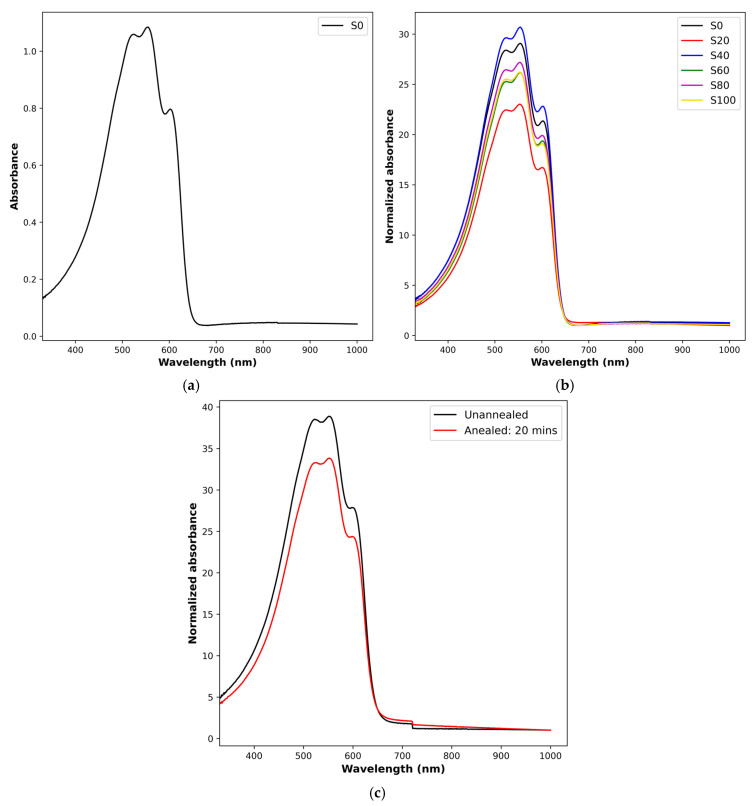
(**a**) Absorbance of as-deposited sample. (**b**) Normalized absorbance of samples annealed at 80 °C at various times. (**c**) Representative spectra from the set of repeated study showing reproducibility of trend.

P3HT is a semi-crystalline material, exhibiting an ordered and amorphous phase, either of which may be dominant according to the material’s molecular weight and processing treatments. The presence of a crystalline phase is usually underscored by a low energy peak or shoulder in the absorbance spectrum, while the amorphous phase contributes significantly to higher energy absorption [20]. The samples studied in the current report have similar qualitative absorbance spectra, typified by distinct shoulders and peaks. An instance of the absorbance is illustrated in Figure 2a for the as-deposited sample S0. The shoulder at 603 nm, which is typical of the crystalline phase, indicates 0-0 vibronic interchain transition, and the maximum at λ = 540 nm represents 0-1 intrachain transition [21,22]. The wavelengths correspond to 2.0 eV and 2.3 eV, respectively, like most reports in the literature. However, in the current case, both peaks are clearly defined. This sharp distinction demonstrates some degree of ordering associated with interchain (0-0) and intrachain (0-1) packing [5]. These features being very pronounced in the unannealed sample can be attributed to the processing procedure, described in the experiment section, which aimed to minimize clustering and encourage ordering, and can provide a visual to the evolution of ordering in a comparative graphical analysis of all samples. The latter is shown in Figure 2b, which revealed the impact of annealing time on the polymer structure through the quantitative values of absorbance at the shoulder and at the higher energy peak. Figure 2b is the normalized spectrum obtained by rationalizing each data set by its smallest value. It allowed for a clearer resolution of the influence of annealing time. The degree of ordering with respect to annealing time is thus revealed in the relative vertical shift in the absorbance spectra with reference to the unannealed sample. Two important pieces of information are revealed here. First, we note that the amorphous state (disorder) dominates higher energy absorption [20], and, as can be seen, the peak shifts with heating time but not in a monotonic fashion. This non-sequential change in magnitude underscores the perturbing influence of annealing time and the sensitivity of structural ordering, similar to temperature-dependent interconversion at a fixed time in [5]. However, unlike the latter, time-dependence is not chronological. In general, there is a downward vertical shift in spectra with respect to the S0 spectrum in agreement with Gurau et al. [12] pertaining to chain ordering. Since the amorphous state is a dominant contributor to an elevated absorbance peak [20], S20—the sample annealed for 20 min—has the lowest spectrum, which we attribute to it having the highest molecular chain ordering and the lowest disordered state. S40 and S0 exhibit the highest and second highest absorbance peaks because of a dominant disordered phase. Second, within the constraint of the 80 °C temperature used here, annealing times > 40 min yielded spectra that lay midway between S0 and S20, indicating an ordered quality with respect to both films. In four different experiments, the absorption spectrum trends were reproducible with changes in magnitudes. An instance is shown in Figure 2c, where, though the magnitudes of absorbance for unannealed and annealed samples differ from S0 and S20 in Figure 2b, respectively, a downward shift is maintained for the annealed sample. Our method of heating multiple samples collectively and extracting samples at specific times informs the conclusion that a single sample heated for an hour goes through several instances of reordering within the hour. For the specific case considered here, the highest ordering was registered at 20 min of annealing.

The energy bandgap of each sample was extracted by detecting the absorption edge and using it in the following formula:

(1) $$E_{gap}\left(\mathrm{eV}\right)=\frac{1242}{\lambda_{ae}}\;\;$$

where $\lambda_{ae}$ is the absorption edge wavelength. Absorption in all the samples starts at 640 nm, yielding $E_{\mathrm{gap}}=1.99\;\mathrm{eV}$, which is consistent with the literature [1,23]. The invariance of E_gap_ with heat treatment time hints on the validity of the idea that annealing only modifies ordering in the molecular chains, which influences charge transport properties. That is, a given temperature fixes the bandgap.

### 3.2. Optoelectronic Properties

**Figure 3:**
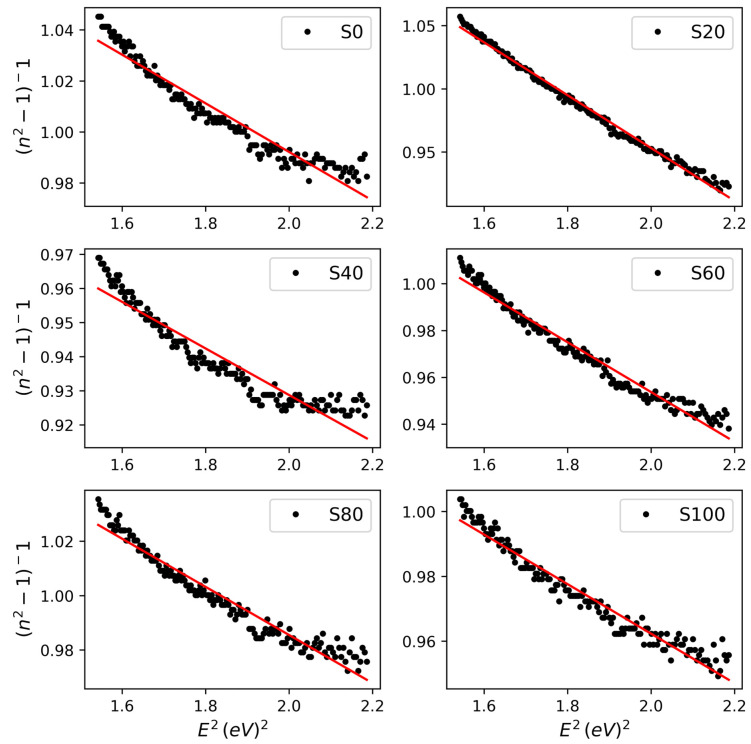
WDD model fits in long wavelength range for confirmation of ordering. Goodness of fit as a measure of quality corroborates inference from absorbance spectra. For S20, $R^{2}=0.991$.

Optoelectronic behavior is directly linked to the structural ordering of materials. To follow up on the probable influence of annealing time on molecular conformity and ordering, we analyzed the refractive index in the frame of the Wemple–DiDomenico (WDD) model and Spitzer–Fan (SF) model in the long wavelength range. In the single oscillator Wemple–DiDomenico model [24].

(2) $$n^{2}=1+\frac{E_{o}E_{d}}{E_{o}^{2}-E^{2}}\;\;$$

where $E_{d}$ is dispersion energy, $E_{o}$ is oscillator energy, and E is photon energy. The oscillator energy represents transition energy from the valence band to conduction band [25] and so can be written as $E_{o}=pE_{g}^{WDD}$, where p is a numeric multiplier whose value indicates the type of transition, and $E_{g}^{WDD}$ is the energy bandgap [25]. In inorganic semiconductors with definitive crystallinity, $E_{g}^{WDD}$ has been in good agreement with bandgaps extracted from the absorption edge or Tauc chart [25]. Therefore, in an analysis of a dual-phase polymer, the existence of a dominant crystalline structure will be quantified by the consistency of $E_{g}^{WDD}$ with bandgaps extracted from absorbance or Tauc plots. Equation (2) was fitted to the refractive indices of the samples, as shown in Figure 3. Sample S20 exhibited a behavior defined by $E_{o}=2E_{g}^{WDD}$, with $E_{g}^{WDD}$ being in same order of bandgap (1.99 eV) from the absorption edge, which underscores the crystalline structure previously deducted from the absorbance analysis (Figure 2). $E_{d}$ is considered to be the energy needed to excite electrons in the conduction band to higher energy levels and indicates dispersive behavior as well as electron–phonon interaction [25]. The implication is that the ease of such a transition rests on the relative magnitude of $E_{d}$. Such ease is apparent in the $E_{d}$ of S20, which explains the high density of states and carrier density in the sample shown in Table 1. One can then suggest that in the frame of the WDD model, $E_{g}^{WDD}\approx E_{g}^{opt}\;$ and low $E_{d}$ indicate crystalline structuring in P3HT. In addition, Figure 3 illustrates the variance of the refractive index with time of exposure to heat treatment.

**Figure 4:**
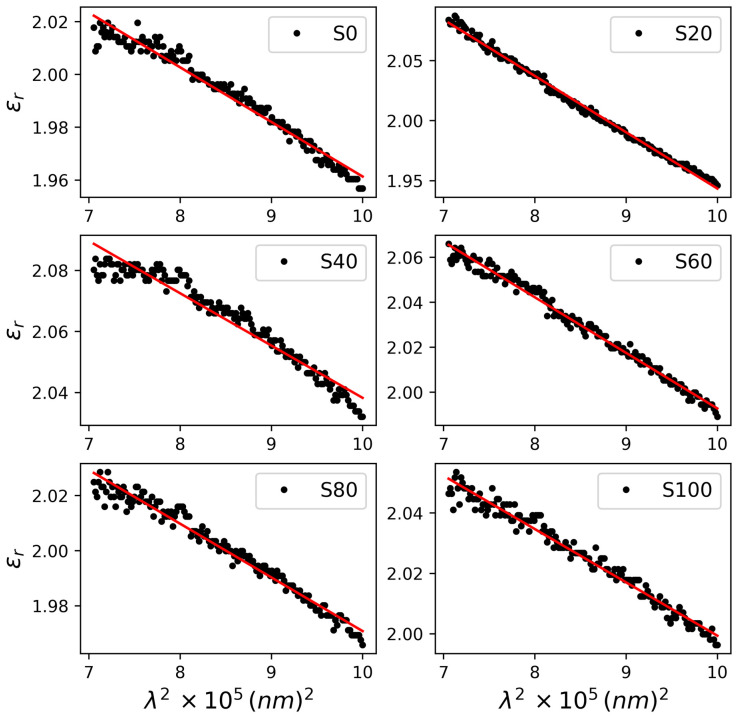
Dielectric analysis using Spitzer–Fan model. The trend of close fit corresponds to absorbance spectra shifts.

**Figure 5:**
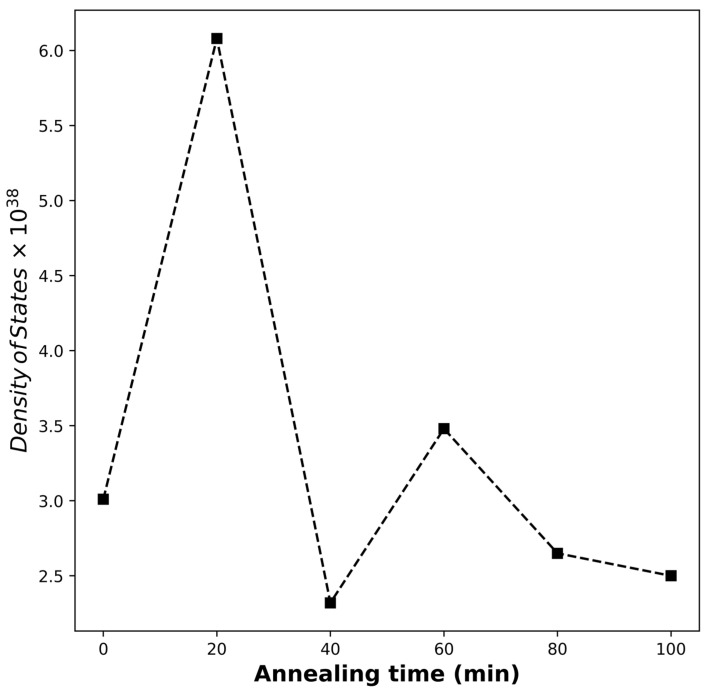
Density of states with respect to annealing time. The value at 20 min affirms the high level of ordering achieved at that interval of time.

The results of the WDD model analysis co-relates with the heat-dependent structural ordering and the interconversion of crystalline and disordered phases in the literature [5]. The trend of close fit of the WDD model to the data clearly follows the vertical shift in absorbance spectra with respect to the spectrum of S0 in Figure 2. It is quantitively validated by the oscillation and dispersion energies of samples S20–S100. A good level of structural ordering encourages high densities of states and carriers. The application of the SF model, in the following form [26]:

(3) $$n^{2}=\varepsilon_{r}=\varepsilon_{\infty }-{\left(4\pi^{2}\varepsilon_{o}\right)}^{-1}{\left(\frac{q}{c}\right)}^{2}\left(\frac{N}{m^{*}}\right)\lambda^{2}$$

permits the deduction of these properties in the long wavelength region. Here, $\varepsilon_{\infty }$ is the dielectric constant at infinity, also known as the lattice dielectric constant, $\left(N/m^{*}\right)$ is the density of states and q and C are the electron charge and speed of light, respectively. It can be seen from Figure 4 that the sensitivity of the real dielectric constants of the samples to Equation (3) is similar to the WDD model, with S20 exhibiting a near-perfect fit for a high-level ordering. From the extracted properties in Table 1, S20 exhibits the highest lattice dielectric constant, which underlines significant phonon contribution and crystalline structuring in agreement with deductions in the WDD model fit. Consequently, this encouraged the highest density of states (DOS) and charge-carrier density. Figure 5 depicts the spread of DOS with annealing time.

## 4. Conclusions

Optoelectronic property profiling of semiconducting polymers within a specific fabrication technique is important to better inform researchers on their applications. A specific demonstration has been performed using the ubiquitous P3HT deposited by spin-coating and annealed at a fixed temperature and variable time within the frame of the absorbance spectrum, Wemple–DiDomenico and Spitzer–Fan models. Our analyses show that molecular chain reordering occurs over the range of time of annealing, showing interconversion of ordered and disordered phases that are underscored by different values of optoelectronic properties. The agreement with the existing literature reveals the importance of the combined Wemple–DiDomenico and Spitzer–Fan models in understanding the electronic structures of polymeric semiconductors. It was found that P3HT processed as described herein achieves the best ordering with the highest density of states and carrier density at 20 min of heat treatment. Thus, it has the potential for an enhanced charge transport, both by itself and with dopants for organic field effect transistor applications. Also, our results demonstrated that a combination of processing method and heat treatment can be leveraged to engineer the electronic properties of P3HT and probably other semiconducting polymers as well.

## Figures and Tables

**Table 1:** Optoelectronic parameters of unannealed (S0) and annealed (S20–S100) samples of P3HT.

Model	WDD Model			SF Model		
Parameter	E_d_ (eV)	E_o_ (eV)	^++^ *ε_o_*	*ε_∞_*	*N*/*m*^∗^ (m^−3^kg^−1^)	** *N* × 10^8^ (m^−3^)
S0	9.33 ± 0.13	11.01 ± 0.17	1.48 ± 0.06	2.37 ± 0.09	3.01 ± 0.15	2.74 ± 0.14
S20	3.81 ± 0.10	5.16 ± 0.16	1.49 ± 0.07	2.62 ± 0.08	6.08 ± 0.16	5.54 ± 0.14
S40	14.10 ± 0.15	14.86 ± 0.10	1.58 ± 0.08	2.27 ± 0.03	2.32 ± 0.086	2.11 ± 0.08
S60	8.30 ± 0.07	9.73 ± 0.14	1.51 ± 0.05	2.40 ± 0.06	3.48 ± 0.16	3.17 ± 0.15
S80	9.98 ± 0.09	11.76 ± 0.16	1.49 ± 0.08	2.38 ± 0.06	2.65 ± 0.12	2.42 ± 0.11
S100	12.10 ± 0.12	13.44 ± 0.10	1.52 ± 0.06	2.28 ± 0.04	2.50 ± 0.14	2.27 ± 0.12

** Calculated using normal electron mass. ^++^ Zero frequency dielectric constant, $\varepsilon_{o}=\;1+\frac{E_{d}}{E_{o}}$.

## Data Availability

Inquiries can be directed to the corresponding authors.
